# Nucleic acid amplification tests in digital microfluidics: the promise of next-generation point-of-care diagnostics

**DOI:** 10.1038/s41378-025-00977-5

**Published:** 2025-08-18

**Authors:** Duc Anh Thai, Yuguang Liu

**Affiliations:** 1 Department of Physiology and Biomedical Engineering, Mayo Clinic, Rochester, MN 55905 USA; 2 Microbiome Program, Center for Individualized Medicine, Mayo Clinic, Rochester, MN 55905 USA; 3 Department of Immunology, Mayo Clinic, Rochester, MN 55905 USA; 4 Department of Surgery, Mayo Clinic, Rochester, MN 55905 USA

**Keywords:** Microfluidics, Electronic devices

## Abstract

Nucleic acid amplification tests (NAAT) have long been used in laboratory facilities and recently revolutionized the field of molecular diagnostics in point-of-care testing. Digital microfluidics (DMF) has emerged as a promising tool to complete the entire NAAT workflow in a miniaturized format with minimum human intervention. Based on electric fields to manipulate independent reaction droplets, the compact DMF system could perform multiple processes simultaneously and automatically in a programmable fashion. This combination is beginning to establish powerful sample-to-answer platforms in remote or resource-limited settings. Herein, we provide a comprehensive overview of the state-of-the-art DMF technology for point-of-care NAAT. This review focused on key principles of DMF platforms and the latest trends in system integration for automated processes of nucleic acid extraction, amplification, and detection. Also, this article discusses current challenges, including control systems, scalability and throughput, as well as future prospects of DMF-based NAAT strategy for the next generation of point-of-care diagnostics.

## Introduction

Disease diagnosis has been conventionally conducted in centralized laboratories or hospitals. Over the past decade, emerging trends have geared toward point-of-care testing (POCT), which does not rely on specialized laboratory equipment or highly trained personnel. These tests can be performed at the sites of accidents, bedsides, homes and remote or resource-limited settings to quickly obtain results for making timely medical decisions^1,2^. Nucleic acid detection is a key aspect in POCT-based diagnosis as these tests can detect DNA and RNA strains (e.g., bacterial, viral strains) with high accuracy and specificity, especially during pandemics and outbreaks. While direct nucleic acid detection can be performed on lateral flow strips (e.g., saliva), detecting low-abundance targets often relies on nucleic acid amplification tests (NAAT). These tests often require multi-step processes to extract the target nucleic acid strains from physiological fluids and amplify them for signal readout^3–5^.

Microfluidics has been increasingly integrated with NAAT, as the microchannels and microstructures in these devices can enable the precise manipulation of small volumes of fluids in a controlled manner. Therefore, microfluidic-based NAAT can perform multiple processes including sample handling, target amplification and signal readout for nucleic acid detections, of which the advantages include rapid analysis, low cost, and ease of operation^6,7^. However, NAAT-based diagnosis often requires extracting targets from complex physiological fluids (e.g., urine, blood), and the amplification of nucleic acids relies on varied types of amplification strategies (e.g., thermal cycling, isothermal) that have different protocols. These cases require a POCT platform that can handle these samples in a more sophisticated manner to perform varied functions.

More recently, digital microfluidics (DMF) has emerged as a new alternative due to its unique ability to handle fluids in a timed and automated manner. In DMF platforms, individual droplets can be manipulated in a programmable fashion on a matrix of planar, individually controlled electrodes without the need for specifically designed microstructures and bulky external setups (e.g., pumps)^8,9^. Multiple processes can be performed simultaneously in a single compact DMF device, requiring minimum human intervention. Therefore, DMF technology has been applied in various lab-on-chip applications including analysis of nucleic acids, proteins and cells as well as disease diagnosis and screening^10^. Besides, various components such as heating elements, biosensors, and imaging systems can be integrated into DMF systems for multiplexed analysis^11–13^. Due to these merits, DMF holds the promise for automated point-of-care molecular diagnostics.

**Fig. 1:**
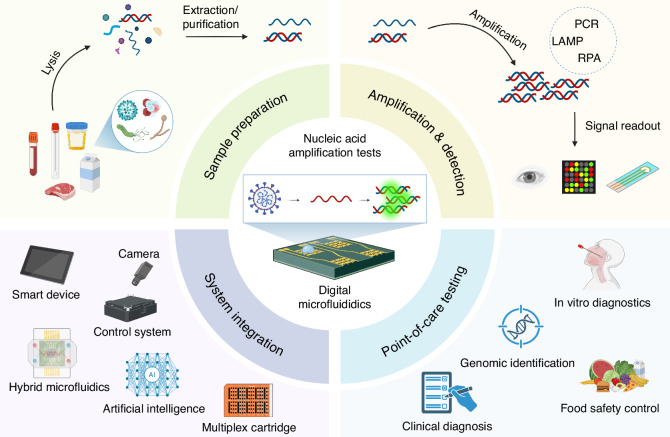
Overview of point-of-care nucleic acid amplification tests in digital microfluidics. The integration of digital microfluidics and peripheral systems automates the entire process of nucleic acid amplification tests (DNA/RNA extraction, amplification, and signal detection) for applications in point-of-care settings. Created in https://BioRender.com

While other reviews provided technical principles and DMF device architectures and designs that support nucleic acid amplification and analysis^11,14^, this review focuses on state-of-the-art DMF technology with an emphasis on NAAT workflow for molecular diagnostics (Fig. 1). Overall, this article summarizes popular techniques for NAAT, key aspects of DMF platforms, and their integration with automated processes of nucleic acid extraction, amplification, and detection. This review also discusses the latest update on commercialization efforts and the integration of DMF technology with artificial intelligence (AI) as well as challenges and prospects.

## Nucleic acid amplification tests

NAAT generally involves processes including sample preparation, amplification, and detection. Typical sample preparation includes cell lysis (e.g., chemical, thermal approaches) followed by nucleic acid extraction and purification^15,16^. The most common amplification is based on thermal cycling such as in polymerase chain reaction (PCR). In fact, PCR is the first NAAT and remains a gold standard for nucleic acid detection. To amplify specific genes, PCR initiates with a pre-denaturation period at 95 °C, then proceeds with 30–40 thermal cycles that consist of three stages: denaturation (95 °C), annealing (50–65 °C), and extension (72 °C)^17^. This methodology has been further developed into, for example, multiplex PCR, quantitative PCR (qPCR), reverse transcription PCR (RT-PCR), and digital PCR to perform varied nucleic acid detection with superb sensitivity^18,19^. Especially, digital PCR methods, including microwell-based digital PCR and droplet digital PCR (ddPCR), can enable precise quantification of the target DNA sequences by calculating the ratio of positive to total microreactions^20,21^.

Nevertheless, PCR often relies on thermocyclers that are typically found in life sciences labs, limiting its broad application in POCT. Alternatively, nucleic acids can be amplified using isothermal amplification strategies. These strategies largely simplify the design and operation of POCT-based NAAT without relying on centralized laboratories and are becoming increasingly popular^22,23^. For example, loop-mediated isothermal amplification (LAMP) has been widely used for nucleic acid amplification in POCT since its discovery in 2000^24^. This reaction uses a set of 4–6 primers that are complementary to distinct regions on specific sequences, offering great selectivity. Instead of the initial thermal denaturation as in PCR, LAMP uses a DNA polymerase with high strand displacement activity to open the double-stranded structure of the target sequence. The LAMP process includes stem-loop formation and exponential amplification and can produce up to a billion copies of the target amplicons in <1 h at a constant temperature (60–65 °C)^25^. Combined with reverse transcriptase, RNA can likewise be detected in the reverse transcription LAMP (RT-LAMP)^26^. In fact, LAMP has been extensively applied in clinical diagnosis, food safety, and environmental monitoring^26–28^.

However, LAMP often requires the design of complex primer sets including “loop primers” for enhanced sensitivity. Therefore, newer amplification technology such as recombinase polymerase amplification (RPA) has gained new attention due to its simplicity in primer design, lowered temperature and rapid reaction. RPA has become one of the most versatile NAAT since it first emerged in 2006^29^. Generally, RPA shares a similar principle with PCR but uses the activity of recombinase under isothermal conditions, and these reactions are often performed at 37–42 °C within only 20–40 min^30^. In fact, RPA is considered as the amplification technique with the shortest turnaround time, and different formats of RPA have been developed including RT-RPA, multiplex RPA, and digital RPA to meet the various needs in disease diagnosis and screening^31,32^. Moreover, RPA ideally combines with the clustered regularly interspaced short palindromic repeats (CRISPR)/Cas in the homogeneous reaction (37 °C), in which the rapid amplification power of RPA is coupled with the highly specific target recognition ability of CRISPR/Cas system, allowing rapid and sensitive detection of specific DNA sequences^33^.

NAAT can be easily coupled with diverse detection methods such as turbidity, fluorescence, colorimetry, and electrochemistry at the endpoint or in real-time^34–38^. To further enhance sensitivity and reduce complexity and amplification biases, various amplification approaches are starting to emerge, such as nucleic acid sequence-based amplification (NASBA)^39^, rolling circle amplification (RCA)^40^, exponential amplification reaction (EXPAR)^41^, and multiple displacement amplification (MDA)^42^. Each methodology possesses its unique characteristics including the amplification mechanisms and reaction conditions.

## Digital microfluidics

DMF is a promising analysis platform in which discrete droplets are formed and manipulated by an actuation force. Several droplet actuation methods have been used in DMF platforms, including electrowetting-on-dielectric (EWOD)^43,44^, magnetic force^45^, surface acoustic wave^46^, and dielectrophoresis^47^. We mainly focus on EWOD-DMF devices because it is the most used mechanism in DMF-based molecular diagnosis^11,14^. Indeed, a wide range of device designs, microfabrication methods, and peripheral systems have been developed for EWOD-DMF devices to meet the requirements of NAAT in point-of-care settings.

### Configurations

**Fig. 2:**
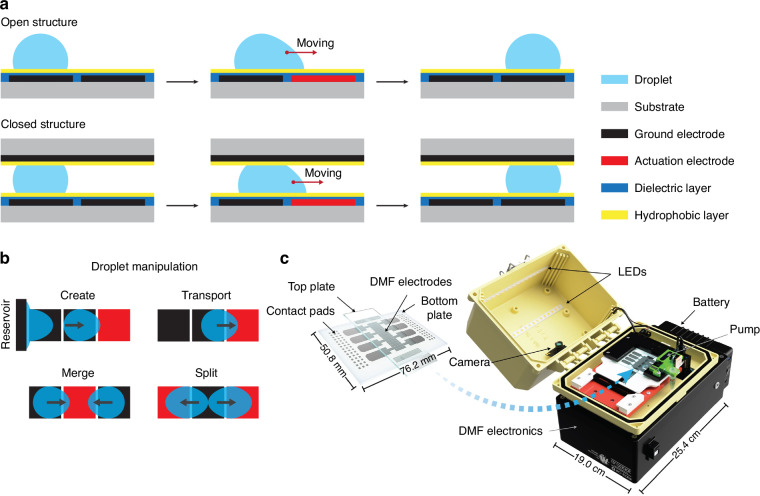
Architecture and principle of digital microfluidics. **a** Configuration and EWOD principle of the open and closed structure in DMF device. **b** Droplet manipulation in EWOD-DMF device: creating daughter droplets from the reservoir, transporting a droplet, merging two or more droplets, and splitting a mother droplet. **c** DMF device and DropBot instrument used in biological studies. Reproduced with permission from ref. ^72^

Overall, DMF platforms have two configurations: an open structure with a single plate and a closed structure with double plates (Fig. 2a). In the open configuration, liquid droplets move on a substrate with an array of actuation electrodes in which ground electrodes are imbedded. In the closed configuration, droplets are sandwiched between a bottom plate patterned with an array of actuation electrodes and a top plate coated with indium tin oxide (ITO)—an optically transparent, electrically conductive material that often serves as the ground electrode^48,49^. While the open DMF devices have reduced complexity and increased adaptability for various applications, droplet handling in these devices is limited to only transport and mixing due to the lack of surface tension required for splitting as in closed DMF devices^44^. Besides, evaporation and aerosol contamination in open DMF devices can be more prominent, limiting their applications in nucleic acid analysis^43^. Therefore, most biological assays were performed in closed DMF systems; this is especially the case when nucleic acid amplification often requires elevated temperatures.

### Design and fabrication

A standard DMF device consists of four key components—substrates, electrodes, dielectric layer, and hydrophobic layer. Glass substrates are the most common because DMF device construction can be streamlined using well-established microfabrication processes. However, in most cases, the number of electrodes on glass-based DMF devices is limited (e.g., most commonly 40–80 electrodes)^13,50^. While silicon-based DMF devices can support a larger number of electrodes—from a few hundred to thousands^51^, these substrates are used to a much lesser extent for point-of-care applications due to the increased costs and complexity of microfabrication. Printed circuit board (PCB) offers a low-cost alternative for batch DMF fabrication. Meanwhile, by imbedding multilayer electrical lines in PCB substrates, the complex layout of connection lines between electrodes and pads in typical glass-based devices can be eliminated^52^. To further lower the cost, flexible substrates such as paper and polyester thin films are starting to emerge for the rapid prototyping of DMF devices for point-of-care applications, especially in resource-limited settings^53,54^. While PCB and flexible substrates excel in cost, rapid prototyping, and scalability, glass and silicon platforms provide superior optical transparency, chemical inertness, and high-resolution microfabrication^9^. The selection of substrates can be guided by specific needs in an application.

Electrodes are primarily fabricated from gold, copper and chromium, and sometimes conductive materials such as ITO due to its optical transparency. In a DMF device, actuation electrodes are often photolithographically patterned on the bottom substrate with ITO coated on the top plate^55^. This typically requires microfabrication by well-trained personnel in clean room facilities, limiting the feasibility of mass production. Therefore, efforts have been directed to develop strategies to build these devices in regular labs. Examples include microcontact printing, screen printing as well as ink-inkjet printing for patterning the electrodes^56,57^. In DMF devices, electrodes are more often designed as interdigitated to enable quick and smooth droplet movement, while droplet manipulation with increased accuracy can be achieved on hexagon and square electrodes^58^.

A dielectric layer is used to insulate the electrodes and build up charges and electric field gradients in the device. The dielectric layer is traditionally made from organic materials (e.g., Parylene and Teflon) and inorganic materials (e.g., silicon oxide and silicon nitride) using various techniques such as vapor deposition, sputtering, vacuum evaporation, and thermal growth^59^. To simplify the processes for dielectric coating, SU-8 and polydimethylsiloxane (PDMS) are sometimes deposited onto the electrodes using spin-coating^60^. The dielectric constant of the material and the thickness of the dielectric layer are key parameters for determining the voltage required for droplet actuation, as governed by the Young–Lippmann equation^48,61^. A thinner dielectric layer can reduce the applied voltage; however, electric leakage and breakdown can sometimes occur, eventually failing the device by electrolysis^62,63^.

In a DMF chip, the hydrophobic coating is used to reduce the surface energy and thus enables electrowetting-based droplet manipulation. Fluoropolymers, including Cytop and Teflon, are commonly used in DMF devices and are often spin-coated or dip-coated on the electrodes. As biofouling is a critical challenge in DMF-based diagnostics, other polymers such as Pluronics can also be an option due to their excellent hydrophobicity and minimal non-specific surface absorption^64^. Alternatively, a common strategy to overcome this obstacle is to fill the device with immiscible oils (e.g., silicone oil), rendering superhydrophobic surfaces, and introducing removable thin films that limits electrode fouling and the evaporation of droplets^65–67^.

### Droplet manipulation

DMF controls individual droplets at the microscale on an array of actuation electrodes in a programmed manner in which droplets are manipulated through the application of voltage to the electrodes based EWOD mechanism^68,69^. Briefly, the applied electric field leads to the change in surface tension and wettability, driving the aqueous droplet from an un-activated electrode (higher surface tension) to the activated electrode (lower surface tension) (Fig. 2a). With the EWOD principle, droplet motion, such as transporting, mixing, and splitting, can be achieved on the planar electrode surface (Fig. 2b)^49,58^. EWOD-based DMF devices have been used for various applications, such as immunoassays, nucleic acid assays for point-of-care diagnostics^14,55,70,71^.

While many research groups built DMF control systems in-house to make DMF technology accessible to a wider community, open-source and commercialized DMF control systems have emerged over recent years. For example, DropBot (Sci-bot Inc.) is a small system that can control the electrodes in DMF devices in a timed and sequenced manner, with precise control of electrostatic driving force and instantaneous drop velocity measurement and fluorescent imaging capability commonly used in biological studies and point-of-care applications (Fig. 2c)^72,73^. Likewise, OpenDrop (GaudiLabs) is another affordable DMF control system, which is equipped with a display, joystick, and buttons to control the complex sequences of droplets in a user-friendly manner, potentially ideal for minimally trained users in point-of-care settings^74^. Future trends include integrated DMF platforms with personal electronics as demonstrated in PortaDrop, a portable, stand-alone DMF system, in which a touchscreen is used to control the droplets^75^.

### Thermal control

**Fig. 3:**
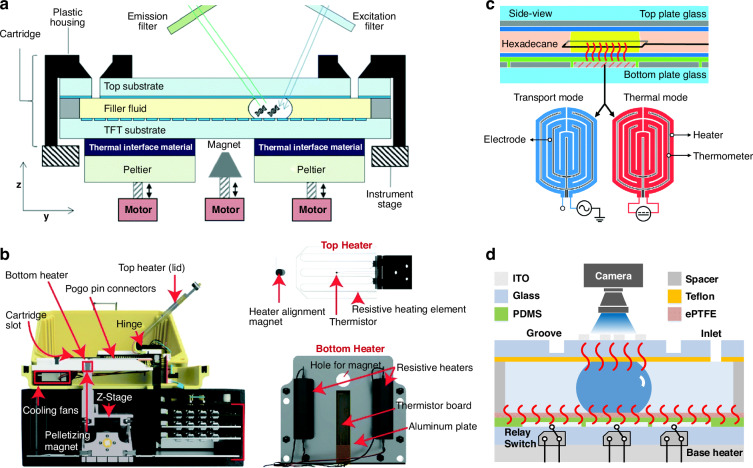
Thermal control in digital microfluidics for nucleic acid amplification. **a** Commercial Peltier element used as an external heating unit in a DMF system. Reproduced with permission from ref. ^76^. **b** A thermal module consisting of top (lid) and bottom (plate) heaters in a DMF system. Reproduced with permission from ref. ^83^. **c** Transport electrode and thermal electrode formed on the same thin-film layer on the surface of the bottom plate of a DMF device achieving droplet actuation (transport mode) and thermal modulation (thermal mode). Reproduced with permission from ref. ^85^. **d** A pincer heating scheme consisting of top and bottom ITO heaters integrated into a DMF chip. Reproduced with permission from ref. ^92^

To implement NAAT in DMF platforms, heating and temperature control modules are essential. Overall, the choice of thermal control strategies varies depending on the device architecture, the thermal profile of the assays, and the specifics of the applications. The use of external heating elements is common in DMF systems. A straightforward way is to simply position a commercial heating element, for example, a Peltier element, under the DMF device to perform tasks such as PCR (Fig. 3a)^76,77^. However, not all commercial heating elements can be directly used with DMF devices, which requires building customized components. For example, aluminum bars can be used as a heater underneath the DMF cartridge, with a thermistor sensor that controls the temperature^78,79^. Likewise, ceramic-based heaters coupled with thin thermocouple sensors can also achieve real-time temperature regulation^80–82^. However, many times, elevated temperature from the bottom of a DMF device can cause condensation on the top plate when handling microscale volumes in DMF-based NAAT. Inspired by benchtop thermocyclers, Narahari et al. developed a thermal module consisting of two independent heating units^83^. The bottom heating unit comprised an aluminum plate and resistive heaters, while the top heater (as a lid) consisted of a resistive wire sandwiched between two sheets of poly(methyl methacrylate) (PMMA) (Fig. 3b). This structure provided the desired temperature and meanwhile minimized temperature gradients, alleviating liquid condensation.

Nevertheless, external heating strategies often have limitations because of high thermal inertia and slow temperature transitions between substrates. To overcome this barrier, a thermal unit can be integrated into the DMF platform to enable rapid heat transfer to the reaction sites. For example, resistors of high thermal conductivity (e.g., platinum) can be patterned on the DMF chip as a heater and temperature sensor for PCR reaction^84^. These resistors can also be integrated with actuation electrodes on the bottom substrate of a DMF device so that droplets can be transported to a thermal electrode which works as a thin-film heater and thermometer (Fig. 3c)^85^. For more precise temperature control, polysilicon resistors can be used due to their tunable temperature coefficient of resistance, especially in the complementary-metal-oxide semiconductor (CMOS) chips^86^. Similarly, microelectrode dot array (MEDA) is a novel technology that can facilitate droplet actuation and meanwhile programmed for different temperature profiles in a CMOS-based DMF, by the use of resistors to control the current passing through the electrodes^87^. Likewise, these strategies are often compatible with PCB-based devices to achieve droplet actuation and thermal modulation at the same time^88,89^. It is worthwhile to note that ITO, which is typically used in DMF devices as a top substrate, is also an excellent candidate for heating and temperature sensing^90,91^. Recently, Wan et al. developed a pincer heating scheme consisting of top and bottom ITO heaters to facilitate rapid temperature transition^92^. In this construct, an active bottom heater maintained a base temperature to suppress both the vertical conductive and horizontal convective heat loss, homogenizing the temperature within a droplet and suppressing bubble formation, while a passive top heater can achieve higher temperatures with low heating power (Fig. 3d). The varied heating strategies hold promise for an all-in-one DMF platform for NAAT at the POCT.

## Nucleic acid amplification tests in digital microfluidics

### DMF-based sample preparation

**Fig. 4:**
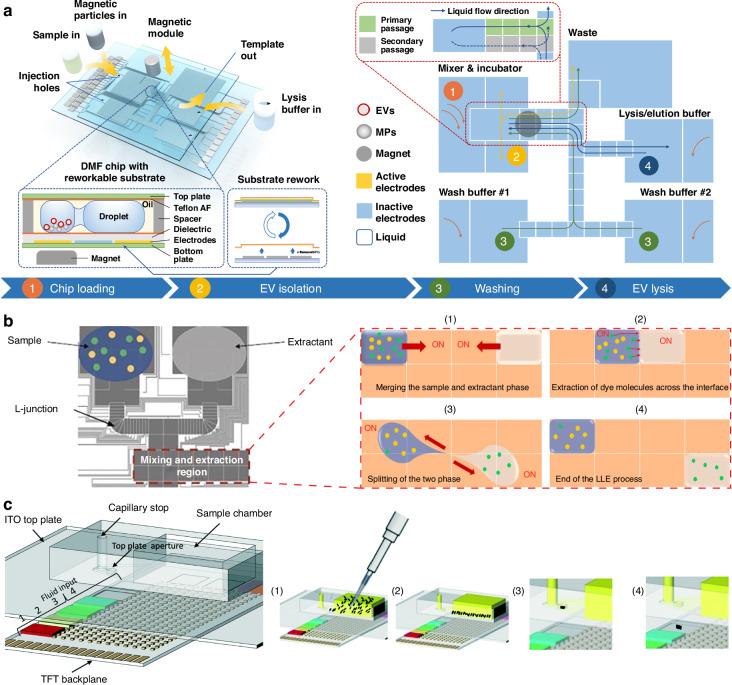
Digital microfluidics for sample preparation. **a** A DMF platform for extracting miRNA from extracellular vesicles (EVs) using solid-phase extraction. Reproduced with permission from ref. ^98^. **b** A DMF platform for extracting DNA from impurities using drop-to-drop liquid-liquid extraction (LLE). Reproduced with permission from ref. ^100^. **c** A DMF platform consisting of a sample chamber to concentrate bacterial DNA from large volumes of urine. Reproduced with permission from ref. ^102^. *MPs* magnetic microparticles

In NAAT, the first and foremost is to extract nucleic acids from complex physiological fluids, often through solid-phase extraction and liquid-liquid extraction. Magnetic bead (MB)-based method is the most popular solid-phase extraction used in the DMF platforms and is typically streamlined with 4 basic steps: cell lysis, DNA/RNA capture, wash, and elution. Briefly, a sample droplet is mixed with lysis buffer to release DNAs/RNAs, which are captured by the MBs. The MBs are washed with droplets with wash buffer and aggregated using a magnet to remove the supernatant. Lastly, a droplet of elution buffer is introduced to elute DNAs/RNAs for the downstream process^78,93,94^. Based on this overall strategy, DMF platforms can be used to extract DNA and RNA from complex body fluids such as whole blood^95,96^. Compared with benchtop methods, these platforms can reduce sample and reagent volumes by >10 times with comparable nucleic acid yields. Newly emerged biomarkers, such as mRNA and miRNA, are also being extracted from cells and extracellular vesicles (EVs) in DMF devices (Fig. 4a)^97,98^. A unique merit of DMF-based target capture is the back-and-forth motions of droplet that can enhance the binding between targets and biorecognition elements by approximately 3 times^99^, which can enable on-chip extraction of small RNAs from cells and EVs within 20–30 min.

On the other hand, liquid–liquid extraction relies on the difference in solubilities of two immiscible liquid phases and is a simpler method for nucleic acid extraction. In DMF devices, this strategy can be implemented by, for example, driving a sample droplet into an immiscible phenolic or ionic solution and cycling back to the air–oil interface to collect DNAs from impurities such as protein molecules (Fig. 4b)^100,101^. Despite the simplicity, liquid–liquid extraction is limited in extraction efficiency and is challenging to process a large volume.

To concentrate the minute amount of genetic materials (e.g., DNA or RNA) from large volumes of fluids (e.g., urine, blood), additional units (e.g., pumps, filters, blockers) can be integrated with a DMF to remove the abundance of non-target components. For example, peristaltic pumps were utilized to drive large-volume blood samples (110–380 μL) for RNA extraction and further purification in microscale volumes (5–15 μL) in the DMF device^96^. To avoid using external pump systems, the sample chamber was designed to concentrate bacterial DNA, for example, *Klebsiella pneumoniae* (*K. pneumoniae*) from 1 mL human urine and scale the sample volume by 500-fold down to a 2 µL droplet prior to downstream processing on the electrode arrays (Fig. 4c)^102^. In addition, the plastic block can be assembled in the DMF device for receiving 1 mL raw samples, performing extraction/purification, and delivering ~50 µL DNA elution^89,103^.

### DMF-based amplification and detection

The second key aspect of NAAT is to amplify the DNAs/RNAs extracted from biological samples to quickly produce sufficient target sequences to enhance the sensitivity of downstream detection. The choice of amplification technologies determines the design and fabrication of DMF devices as well as the integration of other modules (e.g., heating, sensing). Here, we review amplification and detection strategies common in DMF-based NAAT.

#### DMF-PCR

**Fig. 5:**
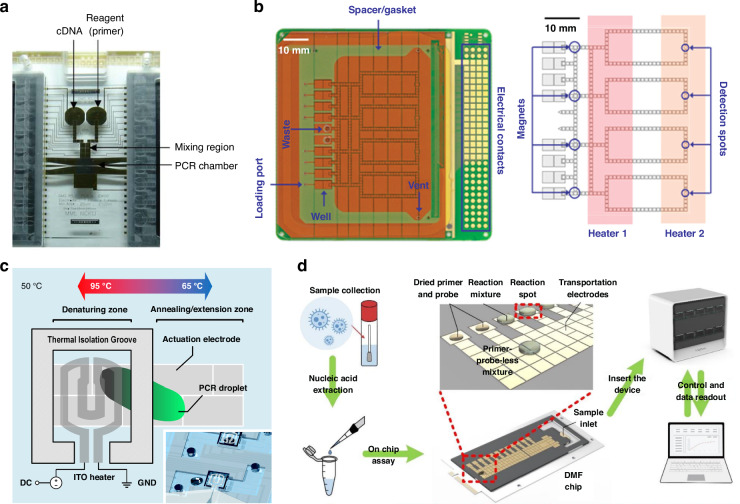
Digital microfluidics for polymerase chain reaction. **a** A DMF platform consisting of a PCR chamber and a single heater providing thermal cycling for DNA amplification. Reproduced with permission from ref. ^84^. **b** A DMF platform consisting of two thermal zones and detection spots for real-time PCR. Reproduced with permission from ref. ^104^. **c** A DMF platform utilizing a thermal isolation groove to reduce the droplet shuttling distance between thermal zones for ultrafast PCR. Reproduced with permission from ref. ^92^. **d** A DMF platform consisting of 12 prestored primer-probe spots for simultaneous RT-qPCR detections of multiple pathogens. Reproduced with permission from ref. ^107^

The first DMF-PCR platform was composed of three main regions including reagent reservoirs, a mixing region and a PCR chamber. A platinum-based heater and temperature sensor were integrated into the PCR chamber along with a controller, providing thermal cycling for DNA amplification (Fig. 5a). With a total sample volume of 15 µL, a gene of Dengue II virus—viral infection typically transmitted through the bites of mosquitos, was amplified in 55 min^84^. Since then, DMF-based PCR garnered increased interest as PCR remains the gold standard for sensitive bacterial and viral detection, yet these tests largely remain inaccessible to populations that need them the most due to the lack of equipment and technical expertise. Therefore, efforts were focused on developing an affordable, all-in-one DMF system to prepare the samples and shuffle the droplets between different temperature zones, such as the denaturation zone (95 °C) and the annealing/extension zone (60 °C). The two-temperature configuration eliminated the transition time between the heating and cooling process, which enabled the bacterial and fungal detection (e.g., methicillin-resistant *Staphylococcus aureus*, *Mycoplasma pneumoniae)* in a 40-cycle real-time PCR within 12 min (Fig. 5b)^78,104,105^. However, performing on-site PCR in DMF devices remains challenging due to the formation of bubbles under high temperatures, and the rapid shuffling of droplets between thermal zones can lead to detrimental dielectric breakdown. To enhance the efficiency and reliability of DMF-based PCR, newer strategies include augmenting horizontal temperature difference and reducing the droplet shuttling distance between thermal zones, for example, using thermal isolation grooves, reducing the reaction time to only 5 min (Fig. 5c)^92^. Other efforts include enhancing detection sensitivity to a single copy per droplet by lowering droplet volumes down to the nanoscale as well as integrating cooling systems (e.g., 4 °C) in DMF platforms^86,106^.

Sometimes, a single nucleic acid target is insufficient for disease diagnosis and screening. Multiplexed NAAT becomes essential, for example, for those who have symptoms of infections but of unknown sources, or the immunocompromised with higher risks of multiple infections. Standard PCR relies on distinct fluorescent colors for multiplexed detection. While this strategy can readily be adopted in DMF platforms, it is limited in the number of targets that can practically be detected (e.g., 3 targets) due to the spectral overlap, which often requires a laborious optimization process to best ensure the outcomes^79^. The unique construct of DMF devices, however, can offer an intrinsic merit of spatial encoding, as different targets can be amplified on electrodes of known locations. A recent example showed the feasibility of a DMF platform for the simultaneous detection of 11 respiratory pathogens from nasal or throat swabs using on-chip RT-qPCR with a sensitivity of 12–150 copies per test and 99.85% accuracy (Fig. 5d)^107^.

#### DMF-LAMP

**Fig. 6:**
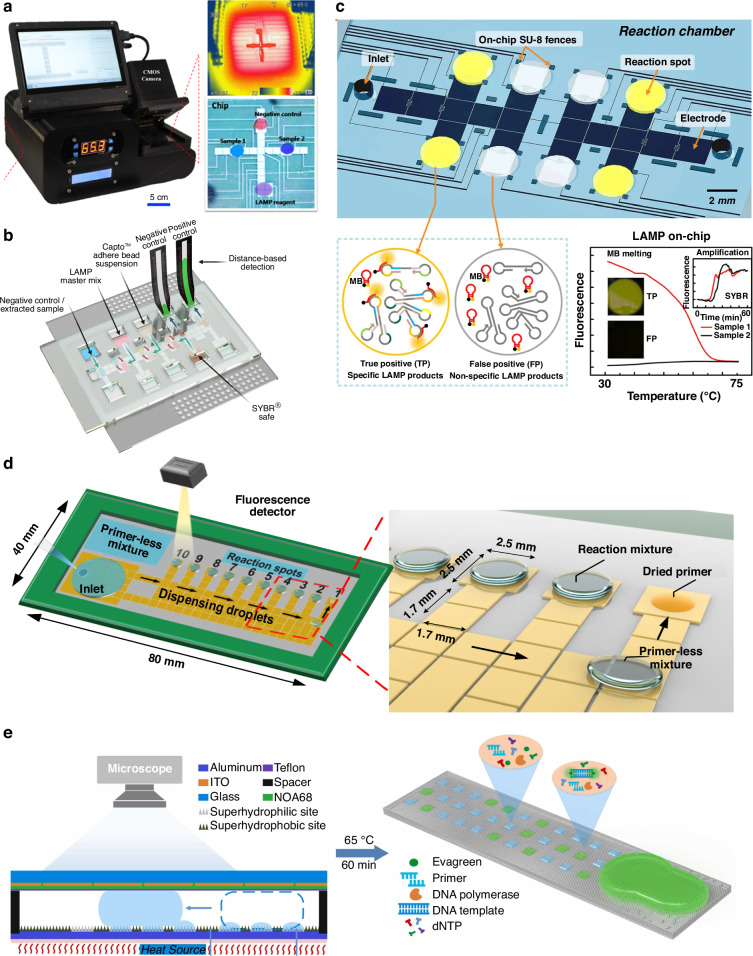
Digital microfluidics for loop-mediated isothermal amplification. **a** A compact instrumental DMF system for fluorescence LAMP detection. Reproduced with permission from ref. ^82^. **b** A DMF-LAMP device integrating with distance-based detection for semi-quantitative readout. Reproduced with permission from ref. ^109^. **c** A DMF-LAMP device utilizing molecular beacon DNA probes for sequence-specific pathogen detection. Reproduced with permission from ref. ^80^. **d** A DMF chip with dried primers for multiplex LAMP detection. Reproduced with permission from ref. ^88^. **e** A DMF device based on superhydrophobic and superhydrophilic micropatterns for digital LAMP reaction. Reproduced with permission from ref. ^77^

Compared with PCR, LAMP can be more straightforward to implement in DMF platforms due to its simple thermal profile, especially in resource-limited settings^90,91^. Many of the strategies discussed earlier can be used to build a compact DMF system that includes electrical circuitry, a temperature regulation system and an detection unit (e.g., fluorescent imaging, photomultiplier tube (PMT))^82,93^ (Fig. 6a). However, these systems are yet to be made widely available, therefore using colorimetric detection instead can be an alternative. For example, LAMP can be used with DNA binding dyes (e.g., SYBR Green) or pH indicators (e.g., neutral red) so that the changes in the color can be detected by naked eyes, with a sensitivity ranging from 10 to 40 copies of DNA per reaction^81,108^. While visual-based detection is challenging for target quantification, a recent effort integrated distanced-based detection to achieve semi-quantification^109^. This is a technique in which analyte amounts are reported based on the length traveled of a visual signal on a patterned paper reporter (Fig. 6b). In this work, the amplified DNAs were mixed with SYBR Safe and transferred to the detection zones. The distance that the dye traveled on the paper strips distinguished the difference in the concentration of salivary SARS-CoV-2 (10^4^ and 10^8^ copies mL^−1^).

However, LAMP mostly relies on indirect detection mechanisms such as turbidity and the use of non-specific dyes, leading to high false-positive rates^110^. Strategies to overcome this drawback include, for example, designing molecular beacon probes that can bind to specific sequences in the LAMP amplicons and discriminating non-specific amplification such as from aerosol contamination. With these optimizations, DMF-based LAMP can detect as low as 10 copies of DNA (e.g., *Trypanosoma brucei*, Coronavirus) per reaction in 15–40 min (Fig. 6c)^80^. Meanwhile, the use of these detection mechanisms makes it intrinsically challenging to implement multiplexed LAMP reactions. However, the construct of DMF offers a unique remedy for this limitation, that is, to spatially encode different targets and simultaneously detect them. For example, Xie et al. developed a DMF chip for multiplex LAMP detection of four foodborne pathogens including *Staphylococcus aureus (S. aureus)*, *Salmonella typhimurium*, *Escherichia coli (E. coli)*, and *Listeria monocytogenes*. Different genes were simultaneously amplified at different spots with dehydrated primers and were fluorescently detected, reducing the need for manually loading the reagents into the device (Fig. 6d)^88^. When combined with colorimetric detection, this platform enables on-site NAAT and detects as low as 10^3^ CFU mL^−1^ of pathogenic bacteria spiked in milk^89^.

Nevertheless, the number of electrodes that can be practically placed in standard DMF devices is limited—up to 120, more commonly 40–80^13,50^, as each electrode is physically connected to a peripheral connector. Therefore, this technology has rarely been used for high-throughput applications such as digital nucleic acid amplification. It is, therefore, essential to form strategies that can accommodate a large number of nanodroplets in areas where LAMP reactions are performed. A potential path forward is illustrated in a recent example where superhydrophobic and superhydrophilic micropatterns were integrated into a DMF device for a digital LAMP reaction. In this platform, reaction droplets were dispensed from the superhydrophobic patterns by EWOD principle to the superhydrophilic micropatterns, where a total of 1818 individual droplets with high uniformity were generated within 1 min (Fig. 6e). This DMF-based digital LAMP enabled absolute DNA quantification with a dynamic range of 1–1000 copies μL^−1^ within 60 min^77^. Meanwhile, future alternatives include the use of active-matrix DMF systems that rely on thin-film transistors (TFTs) to manipulate liquid droplets. In brief, each pixel contains active transistors that act as switches and can be independently addressed by row and column driver lines, supporting tens of thousands of pixels (e.g., 256 × 256)^111,112^. As TFTs can be microfabricated using well-established thin-film processes, making active-matrix DMF devices scalable for mass production.

#### DMF-RPA

**Fig. 7:**
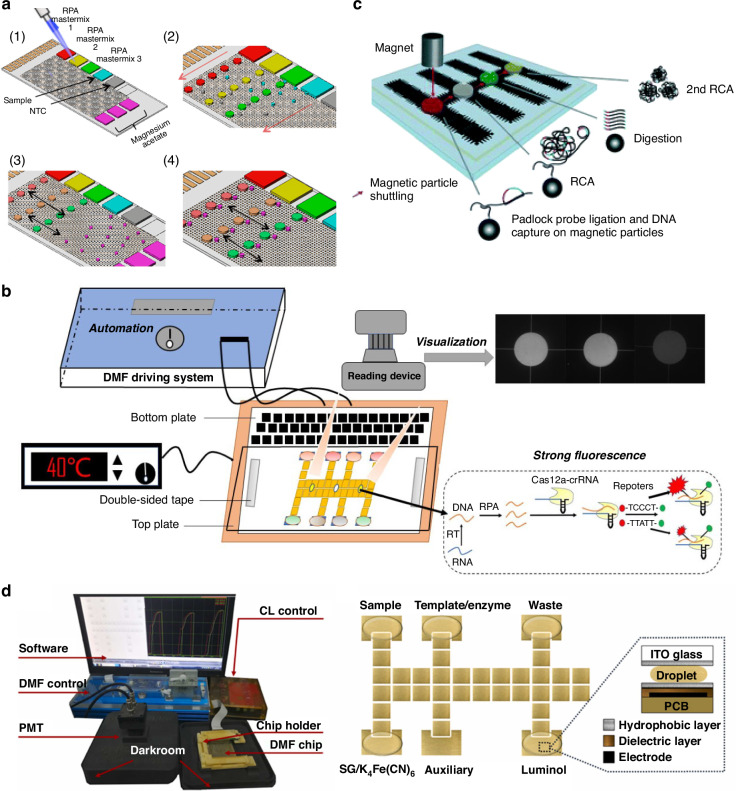
Digital microfluidics for other isothermal nucleic acid amplifications. **a** Numerous RPA reaction droplets manipulated on an active-matrix DMF device for simultaneous detection of multiple genes. Reproduced with permission from ref. ^114^. **b** A DMF device combined with RPA-Cas12a system for automation, specificity, and sensitivity nucleic acid detection. Reproduced with permission from ref. ^115^. **c** Padlock probe ligation and two rounds of RCA performed in a DMF device for single molecule detection. Reproduced with permission from ref. ^119^. **d** A DMF system integrated with EXPAR and chemiluminescence (CL) analysis for automated detection of DNA. Reproduced with permission from ref. ^122^

While LAMP has several distinct merits, the design of primers can be complex, which often constrains the target sequence selection, resolution, and specificity. RPA has been gaining new attention due to its reduced complexity in primer design and ability to achieve sensitive and specific detection at a lower temperature within a shorter time and, therefore, has recently been integrated into the DMF platform. For example, Kalsi et al. implemented an active-matrix DMF device consisting of a TFT backplane (96 × 175 electrodes) that can independently control numerous RPA reaction droplets (at 39 °C) with volume down to 45 nL for fluorescently detecting antimicrobial resistance. The method enables the detection of bla_CTX-M-15_ resistance gene from *E. coli* with a sensitivity of a single copy <15 min^113^. A key advantage of using active-matrix DMF is its large scalability that can lead to programmed, simultaneous detection of multiple genes, allowing for the use of a universally designed active-matrix DMF device for a wide range of specific multiplexed detection based on demands, with enhanced capacity for refined processing body fluids (Fig. 7a)^114^. For example, this platform was used to detect antimicrobial resistance in pre-concentrated *K. pneumoniae* spiked in human urine^102^, with a detection limit of 10^4^ CFU mL^−1^.

However, bacterial or viral loads in infections are often in the range of tens of CFU mL^−1^ (with acute cases being >100 CFU mL^−1^), and a vast number of commensal microbes are present in our body fluids (e.g., saliva, urine). Therefore, it is essential to increase the specificity and sensitivity of NAAT to diagnose or screen for diseases. More sensitive detection mechanisms such as CRISPR can be used along with RPA to recognize specific sequences of RPA products. For example, a RPA-Cas12a-based DMF platform was recently developed for detecting influenza virus and SARS-CoV-2 in nasopharyngeal swab samples, as well as bacterial pathogens in urine and milk (Fig. 7b)^115^. The combined use of RPA and CRISPR/Cas12a largely enhanced the selectivity against competing pathogens due to their intrinsic characteristics, making it promising for DMF-based NAAT in point-of-care settings^116^.

#### Other DMF-based isothermal nucleic acid amplifications

Each amplification mechanism has its strengths and limitations. Determining an amplification strategy that fits the most requires understanding the specific needs (e.g., sample and target types, sample prep) in an application and resources available (e.g., system requirements). For applications that require detecting single-base mismatches in specific DNA sequences especially when the targets are low-abundance, RCA—an isothermal enzymatic process that uses a circular DNA template to exponentially amplify a specific DNA sequence, can be an ideal candidate^117,118^. Overall, the integration of RCA into DMF devices is relatively straightforward as the reactions can be performed at ambient temperature, and the automated droplet handling in DMF can enable multiple rounds of RCA to further enhance sensitivity. Combined with padlock probe ligation, Kuhnemund et al. performed two rounds of RCA on synthetic *Pseudomonas aeruginosa* DNA and achieved a superb detection limit of 1 aM (Fig. 7c)^119^. Unlike RCA which generates long, single-stranded DNA, EXPAR^120,121^, another isothermal amplification technique rapidly amplifies short cDNA sequences, can produce a large number of double-stranded DNA at 37 °C in a DMF device and enables sensitive detection of, for example, HIV, in the range of 10 pM to 2 nM (Fig. 7d)^122^. While both RCA and EXPAR are often used for amplifying DNA, detecting low-abundance RNA targets (e.g., viral RNA) in point-of-care settings can leverage the merits of NASBA^123,124^. This isothermal RNA amplification strategy is less prone to DNA contamination, and has been shown to amplify Zika viral RNA at 41 °C in a DMF device for 90 min^83^. However, NASBA has limited capacity for multiplexing and specificity. These limitations can be remedied by spatially encoding different reactions on different electrodes in DMF devices and further enhancing the purity of the extracted target RNA.

### Integrated DMF-NAAT platform

So far, DMF platforms have been integrated with various components to streamline multiple processes for point-of-care NAAT. While DMF devices are well-suitable for processing microscale volumes (e.g., 1–10 μL), real-world applications sometimes require the sensitive detection of low-abundance targets in a relatively large volume (e.g., urine). To overcome this barrier, pre-concentration units can be integrated into DMF devices to concentrate the targets prior to the NAAT processes. For example, Kalsi et al. interfaced a DMF device with a PMMA-based pre-concentration unit to concentrate *K. pneumoniae* DNA spiked in human urine for RPA reaction and real-time fluorescence detection. The system demonstrated a sensitivity of 10^4^ CFU mL^−1^ in a nearly complete procedure in approximately 30 min, allowing for the detection of antimicrobial resistance genes directly from urine^102^.

**Fig. 8:**
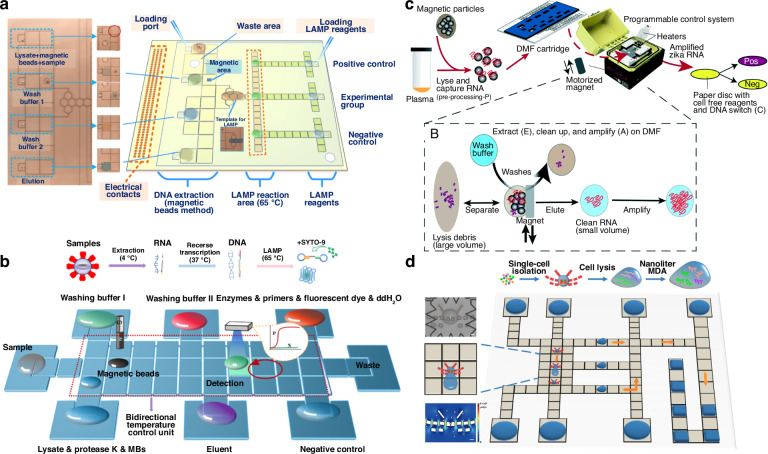
Integrated digital microfluidics for nucleic acid amplification tests. **a** An all-in-one DMF system integrated with magnetic bead-based extraction, LAMP, and real-time signal monitoring for automatic molecular diagnosis. Reproduced with permission from ref. ^93^. **b** A DMF chip combined with a dual-mode thermal control module for RT-LAMP detection of viral RNA. Reproduced with permission from ref. ^125^. **c** A handheld DMF platform (Zed Box) for portable, automated, and integrated Zika viral RNA extraction and amplification. Reproduced with permission from ref. ^83^. **d** A DMF platform with butterfly structure for single-cell immobilization, alkaline lysis, MDA, and gene analysis. Reproduced with permission from ref. ^126^

While distinct modules and functionalities (e.g., thermal, magnetic) have been integrated into DMF platforms to streamline on-chip NAAT processes, more often, the off-chip setups are independent, which adds complexity in handling the platform, especially by those who are minimally trained. Therefore, efforts have been directed to create an all-in-one system, as exemplified by a DMF system developed by Hu et al. The system comprised a microcontroller, magnetic modules, heater, and PMT module for optical detection, which were operated by control software in a compact unit, that can perform sequentially automated MB-based extraction, LAMP, and fluorescence detection (Fig. 8a). The entire on-chip workflow, from nucleic acid extraction from 5 µL of *E. coli* samples to detection, was completed within 60 min^93^. Moreover, nucleic acids, especially RNA, can be highly unstable at ambient temperature or above due to its inherent chemical structure, and therefore cooling mechanisms (e.g., ice) have also been integrated into DMF platforms to better ensure its quality for downstream analysis. Liao et al. combined a dual-mode thermal control module with a PCB chip to provide low temperature (4 °C) and high temperature (37 and 65 °C) for RT-LAMP detection of viral RNA (Fig. 8b). In this system, all steps of NAAT for pseudoviruses of SARS-CoV-2 and monkeypox were completed within 40 min in real-time fluorescence monitoring^125^. To further miniaturize the overall footprint, Zed Box, a handheld DMF platform that contained a programmable control system, heater and motorized magnets, was developed for detecting Zika virus^83^. The self-contained box can be conveniently carried into the field. The entire DMF workflow included sample lysis, RNA extraction and clean-up using MBs, NASBA, and colorimetric detection using a paper-based cell-free protein expression assay, which detected as low as 10^3^ copies mL^−1^ and 100 PFU mL^−1^ Zika viral RNA in spiked human plasma and samples containing Zika virus particles, respectively (Fig. 8c).

While most of the DMF-based NAAT is used for rapid infection or disease screening, these processes can be adapted for environmental testing or food safety monitoring. For example, Zheng et al. used swabs to sample shrimps that were spiked with *Vibrio parahaemolyticus*, a bacterium typically found in seawater that can cause gastroenteritis, and performed NAAT and detected 230 CFU g^−1^ targets using a compact DMF-LAMP system, in just 20 min. Powered by a tailored battery, the build-in instrumentation included an actuation module, temperature controller, CMOS camera, and mini-PC, proving an excellent portability for on-site testing^82^. Similarly, DMF-based NAAT was also used to detect *S. aureus* in milk samples. In this study, immunomagnetic beads were used to capture *S. aureus* from samples (for 20 min) and DNA was extracted at elevated temperature^116^. RPA was then performed to amplify the target sequence (NUC gene) at 37 °C for 20 min, and the use of CRISPR/Cas12a-based detection achieved excellent sensitivity of 32 CFU mL^−1^.

Beyond these examples, DMF technology has also emerged as a new tool for NAAT in single cells^97,126–128^. A key aspect of analyzing single cells in DMF devices requires the isolation of single cells without interfering with the electrowetting functionality. A straightforward way is to integrate microstructures in electrode arrays to contain a single cell in nanoliters of fluid. For example, Rival et al. integrated walled structures to isolate single human cells in 64 nL droplets, and extracted mRNA on MBs for qRT-PCR on-chip. The different processes were streamlined and automated, the entire process was completed in 90 min with single-cell resolution^97^. However, conventional traps such as these can expose trapped cells to damaging stress. Alternatives include the integration of hydrodynamic traps to retain single cells under the hydrophilic surface energy traps rather than physical structures. For example, wettability-based, butterfly-structured hydrodynamic traps were integrated into a DMF array to automatedly and efficiently capture single cells. Unlike in microstructure-based single-cell isolation, this approach enabled selective isolation of the desired single cells by reversely flushing, if necessary. The processes streamlined on-chip include cell immobilization, alkaline lysis, MDA, and gene analysis (Fig. 8d)^126^.

### Hybrid microfluidics

**Fig. 9:**
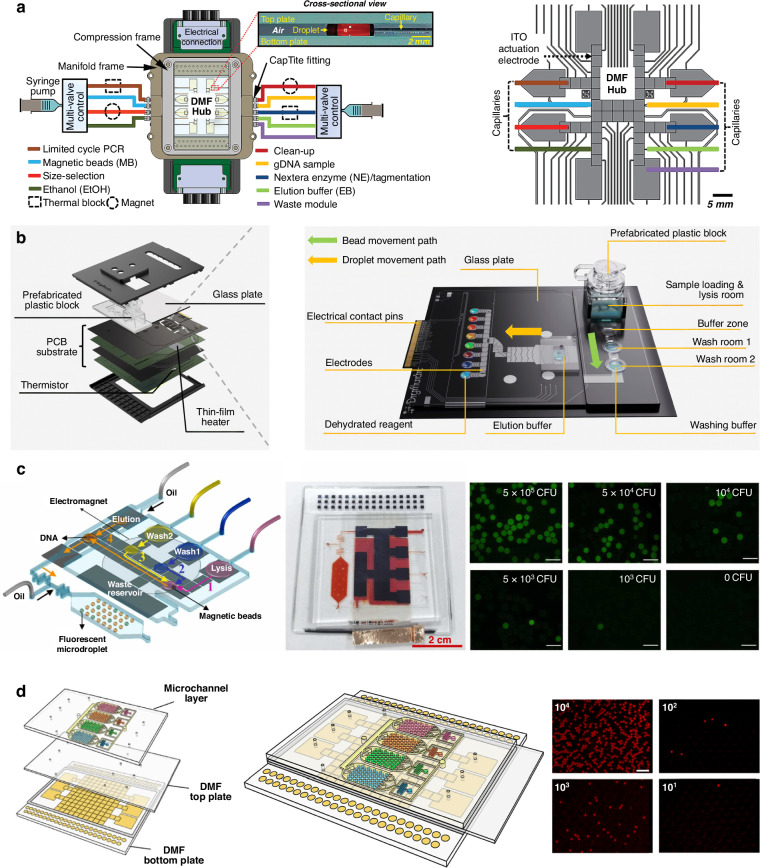
Hybrid microfluidics for nucleic acid amplification tests. **a** A DMF-capillary interface for sample preparation and PCR. Reproduced with permission from ref. ^131^. **b** A macrochannel-to-digital microfluidic platform comprising a plastic macrochannel block for nucleic acid extraction and a DMF for RT-qPCR analysis. Reproduced with permission from ref. ^103^. **c** A photofabricated channel-digital microfluidic chip for nucleic acid extraction and droplet digital PCR. Reproduced with permission from ref. ^94^. **d** A digital-to-droplet microfluidic device comprising a DMF module at the bottom for extraction of nucleic acid and a droplet microfluidics module at the top for ddLAMP. Reproduced with permission from ref. ^133^

To broaden the use of DMF platforms for NAAT and other applications, an innovative strategy is to combine channel-based microfluidics and DMF, establishing hybrid channel-digital microfluidic devices that inherit their unique advantages and meanwhile overcome their limitations. Hybrid microfluidics can add new sample processing, such as sample introduction and chemical separations in microchannels integrated into DMF devices^129,130^. Based on this concept, Kim et al. connected a central DMF hub to the microfluidic capillaries with a multi-valve controller that can load multiple samples and reagents through a syringe pump into the DMF hub in a more precisely controlled manner for sample preparation and PCR (Fig. 9a)^131^. To minimize the need for external pumps, an alternative is to store reagents on-chip, such as in a macrochannel-to-digital microfluidic platform, which consists of a plastic-based macrochannel block for reagent loading and a series of chambers for DNA extraction and purification^103^. The processed elution droplet can then move to the array of electrodes and be mixed with pre-stored reagents for RT-qPCR (Fig. 9b), with the ability to detect up to 32 targets^89^.

Moreover, there exist highly sensitive technologies for NAAT, such as microwell-based digital PCR or ddPCR that provide absolute quantification of nucleic acids^20,21^, streamlining upstream sample preparation with detection in a single device remains difficult. To address these challenges, He et al. created a photofabricated channel-digital microfluidic chip that can perform sample preparation (e.g., extraction, lysis, wash, elution) and transport the purified DNA into microfluidic channels and generate microdroplets at T-junction for ddPCR^94^, enabling the detection of *Salmonella* (10^3^–10^5^ CFU mL^−1^) (Fig. 9c). This concept was then expanded to digital isothermal amplification, especially droplet digital LAMP (ddLAMP)^132^. Recently, Xie et al. implemented a digital-to-droplet microfluidic device that comprised a DMF module at the bottom for sample preparation and a droplet generator microchannel layer at the top for parallel droplet generation and ddLAMP reactions (Fig. 9d)^133^. The entire process was completed in 75 min, and the detection of four pathogens from clinical specimens achieved 91.1% consistency compared with conventional methods. Table 1 highlights the key features and comparisons of several DMF-based NAAT strategies for point-of-care diagnostics.

**Table 1:** Summary of several digital microfluidics for nucleic acid amplification tests

System architecture	Sample preparation	Amplification	Detection	Total time and sensitivity	Target	Ref.
• Glass chip • Single platinum heater	N/A	PCR (95, 53, and 72 °C, 55 min)	Off-chip electrophoresis (Ethidium bromide)	5 ng µL^−1^ (cDNA)	Dengue II virus	^84^
• PCB chip • Two aluminum heaters • Miniature fluorimeter	N/A	qPCR (95 and 60 °C, 12 min)	Real-time fluorescence (EvaGreen)	1 copy reaction^−1^	MRSA and *C. albicans*	^78^
• PCB chip • Two aluminum heaters • Four-channel fluorimeter	N/A	Multiplex qPCR (95 and 60 °C, 18 min)	Real-time fluorescence (TaqMan)	1 copy reaction^−1^	MRSA, *M. pneumoniae*, and *C. albicans*	^79^
• CMOS chip • Three polysilicon heaters • SPAD module	N/A	qPCR (94, 72, and 60 °C, 60 min)	Real-time fluorescence (EvaGreen)	1 copy droplet^−1^	*S. aureus*	^86^
• PCB chip • Four thermoelectric heaters	N/A	PCR (95, 50, 68, and 4 °C, 58 min)	Off-chip electrophoresis (Ethidium bromide)	250 pg μL^−1^	Bacteriophage M13mp18	^106^
• Glass chip • Top and bottom ITO heaters • Fluorescence microscope	N/A	qPCR (95 and 65 °C, 3.7 min)	Real-time fluorescence (EvaGreen)	10 copies reaction^−1^	HIV-1 *gag* and Human *HEXA* gene	^92^
• PCB chip • Two aluminum heaters • Miniature fluorimeter	Off-chip MB-based DNA extraction	qPCR (95 and 58 °C, 60 min)	Real-time fluorescence (TaqMan)	100 CFU mL^−1^ (98% sensitivity)	*M. pneumoniae* in respiratory specimens	^104^
• PCB chip • Two aluminum heaters • Miniature fluorimeter	Off-chip column-based DNA extraction	qPCR (95 and 58 °C, 48 min)	Real-time fluorescence (Uracil-DNA glycosylase)	100 CFU mL^−1^ (94% sensitivity)	*C. albicans* in blood	^105^
• PCB chip • Resistive thin-film heater • Fluorescence detector	Off-chip column-based RNA extraction	RT-qPCR (50, 95, and 60 °C, 40 min)	Real-time fluorescence (TaqMan)	12–150 copies test^−1^ (99.85% sensitivity)	11 respiratory pathogens in nasal or throat swabs	^107^
• Silicon chip • Magnetic module • Single Peltier heater • Fluorescence camera	MB-based mRNA extraction (35 min)	RT-qPCR (50, 95, and 60 °C, 55 min)	Real-time fluorescence (FAM)	• 90 min • 1 cell droplet^−1^ (10 pg mRNA)	*Myc* gene in human cells	^97^
• TFT chip • Magnetic module • Two Peltier heaters • CMOS camera	MB-based RNA extraction (90 min)	RT-qPCR (50, 95, and 57 °C, 100 min)	Real-time fluorescence (TaqMan)	• 190 min • 1 copy droplet^−1^	SARS-CoV-2 in saliva	^76^
• PCB-PC chip • Magnetic module • Resistive thin-film heater • Photodiode detector	Plastic block pre-concentration, MB-based RNA extraction (20 min)	RT-qPCR (50, 95, and 60 °C, 100 min)	Real-time fluorescence (TaqMan)	• 120 min • 15 copies test^−1^ (100% sensitivity)	SARS-CoV-2 and Flu A/B in nasopharyngeal swabs	^103^
• Glass-PDMS chip • Syringe pump • Electromagnet • ITO film heater • Fluorescence microscope	MB-based DNA extraction (60 min)	ddPCR (95 and 60 °C, 60 min)	Fluorescence (FAM)	• 120 min • Quantification (0–10^5^ CFU mL^−1^)	*Salmonella*	^94^
• Glass chip • ITO film heater	N/A	LAMP (65 °C, 45 min)	Off-chip electrophoresis (Ethidium bromide)	0.5 ng µL^−1^	*c-Myc* oncogene	^90^
• Glass chip • PTC heater • Fluorescence microscope	N/A	LAMP (65 °C, 40 min)	Real-time fluorescence (Molecular Beacon)	10 copies reaction^−1^	*T. brucei*	^80^
• Glass chip • PTC heater	N/A	LAMP (65 °C, 60 min)	Colorimetry (SYBR Green I)	40 copies reaction^−1^	*T. brucei*	^81^
• Glass chip • ITO film heater • Fluorescence microscope	N/A	LAMP (59.5 °C, 90 min)	Real-time fluorescence (EvaGreen)	0.5 ng µL^−1^	c-*Myc* oncogene	^91^
• MEDA-CMOS chip • MEDA heater • Microscope	N/A	RT-LAMP (65 °C, 30 min)	Turbidity (Mg_2_P_2_O_7_)	N/A	SARS-CoV-2	^87^
• Aluminum-glass chip • Peltier heater • Fluorescence microscope	N/A	ddLAMP (65 °C, 60 min)	Fluorescence (EvaGreen)	Quantification (1–1000 copies µL^−1^)	λDNA	^77^
• PCB chip • Thin-film heater • Fluorescence microscope	Off-chip column-based DNA extraction	LAMP (57 °C, 40 min)	Real-time fluorescence (SYBR Green I)	10^3^ CFU mL^−1^	*E. coli*, *S. aureus*, *S. typhimurium*, and *L. monocytogenes*	^88^
• PCB chip • Resistance-wire heater	Off-chip column-based DNA extraction	LAMP (63 °C, 30 min)	Colorimetry (Neutral red, 25 °C, 2 min)	10 copies µL^−1^	EHP, IHHNV, and WSSV	^108^
• Glass chip • Resistive heater • Paper strip sensor	Off-chip column-based RNA extraction	RT-LAMP (65 °C, 40 min)	Distance-based detection (SYBR Safe, 25 °C, 5 min)	Semi-quantitation (10^4^ and 10^8^ copies mL^−1^)	SARS-CoV-2	^109^
• Glass chip • Ceramic heater • CMOS camera	Q-tip sampling, NaOH-based DNA extraction (3 min)	LAMP (65 °C, 18 min)	Real-time fluorescence (SYBR Green I)	• 21 min • 230 CFU g^−1^	*Vibrio parahaemolyticus* in spiked shrimps	^82^
• PCB chip • Magnetic module • PTC heater • PMT module	MB-based RNA extraction (23 min)	LAMP (65 °C, 35 min)	Real-time fluorescence (FAM)	• 60 min • 10 copies µL^−1^	*E. coli* and SARS-CoV-2	^93^
• PCB-plastic chip • Magnetic module • Polyimide heater	MB-based DNA extraction (30 min)	LAMP (62 °C, 30 min)	Colorimetry (Neutral red, 25 °C, 1 min)	• 60 min • 10^3^ CFU mL^−1^	*E. coli* and *S. aureus*	^89^
• PCB chip • Electromagnet • Thermoelectric heater • Fluorescence detector	MB-based RNA/DNA extraction (10 min)	RT-LAMP (37 °C, 5 min; 65 °C, 25 min)	Real-time fluorescence (SYTO-9)	• 40 min • 10 copies µL^−1^	SARS-CoV-2 and Monkeypox	^125^
• Glass-PDMS chip • Magnetic module • Syringe pump • Polyimide heater • Fluorescence microscope	MB-based DNA extraction (15 min)	ddLAMP (65 °C, 60 min)	Fluorescence (Cy5)	• 75 min • Quantification (10–10^4^ copies µL^−1^)	*E. coli, P. aeruginosa, K. pneumoniae*, and *E. faecalis*	^133^
• TFT chip • ITO top heater • Fluorescence microscope	Off-chip column-based DNA extraction	RPA (39 °C, 15 min)	Real-time fluorescence (Exo-probe)	1 copy reaction^−1^	*bla*_*CTX*-M-15_ gene from *E. coli*	^113^
• TFT chip • Built-in heater • Digital camera	Off-chip column-based DNA extraction	RPA (39 °C, 25 min)	Real-time fluorescence (Exo-probe)	10 copies reaction^−1^	bla_CTX-M-15_, bla_KPC_, and bla_NDM-1_ gene from *E. coli*	^114^
• Glass chip • Heating plate • Fluorescence microscope	Off-chip column-based RNA extraction	RT-RPA (37 °C, 10 min)	Fluorescence (CRISPR/Cas12a, 37 °C, 20 min)	10 copies reaction^−1^ (100% sensitivity)	Flu A/B and SARS-CoV-2 in nasopharyngeal swabs	^115^
• TFT-PMMA chip • Magnet bar • Built-in heater • Digital camera	Pre-concentration, MB-based DNA extraction (15 min)	RPA (39 °C, 15 min)	Real-time fluorescence (Exo-probe)	• 30 min • 10^4^ CFU mL^−1^	*K. pneumoniae* in spiked urine	^102^
• Glass chip • Magnet bar • Heating plate • Fluorescence microscope	MB-based DNA extraction (25 min)	RPA (37 °C, 20 min)	Fluorescence (CRISPR/Cas12a, 37 °C, 10 min)	• 55 min • 32 CFU mL^−1^	*S. aureus* in urine and milk	^116^
• Glass chip • Heating plate • Fluorescence microscope	N/A	RCA (37 °C and 65 °C, 60 min)	Fluorescence (Padlock probe, 75 °C and 55 °C, 12 min)	1 aM reaction^−1^ (synthetic DNA)	*P. aeruginosa*	^119^
• PCB chip • Graphene film heater • PMT module	N/A	EXPAR (37 °C, 45 min)	Chemiluminescence (SYBR Green/ferrocyanide/luminol, 25 °C, 10 min)	4.5 pM reaction^−1^ (cDNA)	HIV DNA	^122^
• Glass chip • Magnetic module • Built-in top and bottom heaters • Paper disc sensor	MB-based RNA extraction (30 min)	NASBA (65 and 41 °C, 90 min)	Colorimetry (Cell-free assay, 37 °C, 175 min)	• 295 min • 100 PFU mL^−1^ (100% sensitivity)	Zika virus in plasma	^83^

*N/A* not applicable, *PC* polycarbonate, *PTC* positive temperature coefficient, *SPAD* single-photon avalanche diodes, *MRSA* methicillin-resistant *Staphylococcus aureus*, *EHP* *Enterocytozoon hepatopenaei*, *IHHNV* infectious hypodermal and hematopoietic necrosis virus, *WSSV* white spot syndrome virus

## Emerging trends and future perspectives

### Commercialization status

While DMF technology has yet to be widely adopted, several commercial products have emerged in the molecular diagnostics market over the past two decades with continued advancements. Earlier, Advanced Liquid Logic (Morrisville, USA), founded in 2004, emerged as a leader in providing electrowetting-based liquid handling solutions, which attracted major industrial companies and was acquired by Illumina (San Diego, USA) in 2013, aiming to deliver hands-free and efficient library preparation workflow for next-generation sequencing^134^. The technology was later licensed to GenMark Diagnostics (founded in 2010, Carlsbad, USA), which developed ePlex®, an integrated system that incorporated DMF technology and their proprietary eSensor® to perform automated multiplexed nucleic acid testing in a single-use cartridge^135^. The ePlex® instrument was approved by U.S. Food and Drug Administration (FDA) and used to detect a wide range of pathogens (e.g., bacterial, viral, fungal) in varied physiology samples including respiratory fluids, blood, and nasopharyngeal swabs^136^. In 2021, Roche (Basel, Switzerland) acquired GenMark to expand its molecular diagnostics portfolio, known as Roche Diagnostics (Indianapolis, USA), and rebranded the platform as cobas® eplex—an automated in vitro diagnostic device that uses single-use test cartridges to perform nucleic acid extraction, target amplification via PCR or RT-PCR, competitive DNA hybridization, and electrochemical detection to fit a wide range of needs in NAAT. Likewise, Baebies (founded in 2014, Durham, USA) expanded the market to newborn and pediatric screening (e.g., SEEKER® and FINDER®) for lysosomal storage disorders. In 2021, Baebies was given Emergency Use Notification by the FDA for FINDER® SARS-CoV-2 Test^137,138^.

Soon after, international efforts emerged to commercialize DMF platforms. For example, Digifluidic (Zhuhai, China, founded in 2018), a spin-out of the University of Macau, has been developing integrated DMF platforms for automated identification of multiple respiratory pathogens, featuring their Virus Hunter series such as Virus Hunter 2.0^103^ and Virus Hunter Plus®^139^. In addition, GEMFluidix (Los Angeles, USA, founded in 2019) specializes developing in DMF Cartridges for SARS-CoV-2 tests with its branch in Taiwan, China^138,140^. Sharp Life Science (Oxford, UK) focuses on scaling the technology and developing active-matrix DMF, also known as aQdrop™ for in vitro diagnostics SARS-CoV-2 at the point of need^76^. Table 2 shows a comparison of commercial digital microfluidic products regarding nucleic acid amplification for in vitro diagnostics.

**Table 2:** Commercial digital microfluidic platforms for nucleic acid amplification tests

Product	Manufacturer	Year	Chip	Testing panel	Analytical performance	Ref.
ePlex® (cobas® eplex)	GenMark Diagnostics (a member of Roche)	2013	PCB	• Respiratory pathogens • Gram-positive pathogens • Gram-negative pathogens • Fungal pathogens	• PCR/RT-PCR, voltammetry • Sensitivity: 250 copies mL^−1^ • Time: 90 min	^135,136^
FINDER®	Baebies	2021	PCB	SARS-CoV-2	• RT-PCR, qualitative fluorescence • Sensitivity: 1000 copies mL^−1^ • Time: 17 min	^137^
Virus Hunter	Digifluidic	2023	PCB	• SARS-CoV-2/FluA/FluB • Viral shrimp diseases • Cat respiratory and zoonotic diseases	• RT-PCR, real-time fluorescence • Sensitivity: 1000 copies mL^−1^ • Time: 90–120 min	^103,139^
qPCR Cartridge	GEMFluidix	2022	PCB	SARS-CoV-2	• qPCR, real-time fluorescence • Sensitivity: 1000–10,000 copies mL^−1^ • Time: 18 min (no extraction)	^138,140^
aQdrop™	Sharp Life Science	2021	TFT	SARS-CoV-2	• RT-qPCR, real-time fluorescence • Sensitivity: 1000 copies mL^−1^ • Time: 190 min	^76^

### Microbial identification

While sequence-specific nucleic acid amplification is more common in NAAT for POCT, it requires a priori suspicion of a known target strain. In medical emergencies where sources of infections are challenging to identify, untargeted detection of, for example, a broad range of microbial species, can be achieved using 16S ribosomal RNA amplification followed by sequencing. Yet this approach is limited to straightforward bacterial classification without generating additional genomic information (e.g., antibiotic resistance). Over the past recent years, whole genome sequencing emerged as a promising alternative for microbial NAAT. DMF platforms proved effective for rapid isothermal DNA amplification, such as MDA, due to its high fidelity and sequence coverage, as well as the feasibility to perform the reactions at near-room temperature^126,128,141^. While MDA can amplify the whole genome or transcriptome of as few as a single bacterial cell in conventional microfluidic devices, it requires overnight to reach an amount of nucleic acid sufficient for downstream sequencing (e.g., next-generation sequencing, Nanopore sequencing)^142–144^. The use of a DMF device to constantly move the droplet during the reaction can speed up the amplification. In fact, in a DMF device, the whole genome of as low as 10 fg of *Corynebacterium glutamicum* DNA was amplified with high purity in <2 h for Nanopore sequencing, paving the way for identifying low-abundance bacterial species in a non-targeted manner in urgent settings^141^.

### Integration with artificial intelligence

As an automated liquid handling technology, DMF platforms can readily leverage AI to create multipurpose, smart DMF devices that can perform on-demand functions in an autonomous manner. First, electrodes in DMF devices can sometimes breakdown during an experiment, and this can impede the rest of the processes to be performed on other electrodes. An ideal solution is to determine alternative droplet routing strategies in real-time based on the electrodes that are functional. For example, artificial neural network detection model can be trained to recognize droplets, determine their size and location^145^. With real-time feedback, this AI-empowered DMF can use reinforcement learning to capture the underlying health conditions of electrodes, adapt to the electrode malfunction, and make online decisions for droplet routing^146^. Further, Kawakami et al. designed a deep reinforcement learning-based routing algorithm to adapt to varied types of errors in DMF experiments, with the ability to manage common errors (e.g., cell degradation, droplet residues) and underlying errors (e.g., electrode breakdown, fluctuations in temperature)^147^.

While AI-powered DMF technology largely remains an un-cut gem in biotechnology, pioneering efforts have been focused on AI-powered imaging, for example, to identify targets of interest and use a feedback loop to control droplet routing on-demand. Lamanna et al. developed DMF functionalities to facilitate automated cell culture, fixation, and the staining of adherent cells, while a custom convolutional neural network was used to identify the location of the cells of interest from heterogeneous populations and direct a high-energy laser to lyse them for downstream processes including MDA and sequencing^148^. Similarly, Guo et al. integrated deep learning image recognition with DMF to move the droplets with the Safe Interval Path Planning algorithm and accurately classify cells within nL droplets using YOLOv8, a state-of-the-art computer vision model, enabling label-free parallel cell sorting based on morphology in DMF^111,149^. Integrating machine learning algorithm with DMF control system can also dynamically adjust dispensing volume, droplet moving rates, and mixing efficiency to ensure precise and reproducible device operations^150^. Future trends include the use of AI to predict experimental parameters, enabling online optimization of experimental conditions (e.g., incubation time, reagents, temperature) to ensure the outcomes^151^. Particularly, AI can optimize PCR parameters, empower result analysis, and even automate various steps in testing processes, ensuring optimal DNA amplification and detection even with complex or compromised samples^152^.

### Challenges and prospects

Despite the advantages of DMF for NAAT, challenges exist. First, standard DMF is limited in throughput for molecular diagnostics due to the limited number of electrodes that can be practically patterned on a substrate. A straightforward solution is to employ different primers, probes, and fluorescence channels to simultaneously analyze multiple target sequences at different locations in a chip^79,88,103,113^. Newer trends gear toward active matrix technology to build TFT-based DMF platforms that allow tens of thousands of electrodes in a single DMF device, with the feasibility of integrating peripheral systems (e.g., heater, thermistor, and sensor) using established thin-film processing^76,112,113,149,153^. While these platforms can be challenging to build in academic research labs, major industry players (e.g., Sharp) are leading the manufacturing of these devices and expanding their use, for example, from display to diagnostics^76,113^. However, for applications that do not require high throughput, the most cost-effective manufacturing options exist, for example, ink-jet printing DMF electrodes on paper-based substrates^53,154,155^.

Second, most of the DMF devices are powered by high voltages, require manually loading samples and reagents and rely on wired data transfer, creating barriers to using these systems for POCT in minimally trained hands, especially in resource-limited settings. To reach a broader impact, first, low-cost triboelectric nanogenerators can be used to provide high voltage, low current output for electrode actuation^156,157^. Meanwhile, pre-storing dehydrated reagents (reaction mixtures, probes, or indicators) on-chip can be an alternative, reducing the need for manual pipetting in NAAT workflow^83,88,108^. In the future, an AI-powered smart DMF system will essentially ensure a fully automated scenario and online experimental programming. The popularity of wireless communications, internet-of-things (IoT), and data clouding allows data transmission with guaranteed security, progressing toward hospital-on-chip for intelligent healthcare industry^158^. In summary, the standardized infrastructure, convenient manufacturing, and versatile control systems will inspire the current ecosystems of DMF to be more accessible to a wide range of users.

## Conclusions

DMF enables the manipulation of individual reaction droplets in a programmable manner based on electric fields. Compared to benchtop methodologies or conventional microfluidics, DMF-based diagnostic tools demonstrated the outstanding precision and automation of NAAT workflow including sample preparation, nucleic acid amplification, and signal analysis. The integration of DMF and ancillary modules builds the all-in-one platforms for point-of-care diagnostics. Academic research has continually contributed to biological, medical, and clinical applications, moving toward the possibility of commercialization. The efforts are anticipated to advance multiplexity, throughput, and system automation to prove the promise of next-generation diagnostics. Also, technical issues and reliability-cost balance should be addressed to make it become widespread adoption. Overall, despite the potential of DMF in molecular diagnostics, aspects such as miniaturization, standardization, and intelligence should be considered to meet the true ideal of a point-of-care test that is accessible to anyone, anywhere, and anytime.
